# New Books

**Published:** 2009-09

**Acute Exposure Guidelines for Selected Airborne Chemicals, Vol. 7**

Committee on Acute Exposure Guideline Levels, Committee on Toxicology, National Research Council

Washington, DC:National Academies Press, 2009. 236 pp. ISBN: 978-0-309-12755-4, $50.75

**Environmental Change and Food Security in China**

Jenifer Huang McBeath, Jerry McBeath

New York:Springer, 2009. ISBN: 978-1-4020-9179-7, $199

**Environmental Health Sciences Decision Making: Risk Management, Evidence, and Ethics**

Yank Coble, Christine Coussens, Kathleen Quinn, eds.

Washington, DC:National Academies Press, 2009. 92 pp. ISBN: 978-0-309-12454-6, $21

**Fundamentals of Patenting and Licensing for Scientists and Engineers**

Matthew Y Ma

Hackensack, NJ:World Scientific, 2009. 292 pp. ISBN: 978-981-283-420-1, $68

**Integrating U.S. Climate, Energy, and Transportation Policies**

Liisa Ecola, Scott Hassell, Michael Toman, Martin Wachs

Santa Clara, CA:RAND Corporation, 2009. 52 pp. ISBN: 978-0-8330-4670-3, $22

**Metallothioneins and Related Chelators**

Astrid Sigel, Helmut Sigel, Roland K.O. Sigel, eds.

New York:Springer, 2009. 514 pp. ISBN: 978-1-84755-899-2, $299

**Real-Time and Deliberative Decision Making**

Igor Linkov, Elizabeth Ferguson, Victor S. Magar, eds.

New York:Springer, 2009. 456 pp. ISBN: 978-1-4020-9024-0, $199.95

**Science in Democracy: Expertise, Institutions, and Representation**

Mark B. Brown

Cambridge, MA:MIT Press, 2009. 368 pp. ISBN: 978-0-262-01324-6, $56

**The Economics of Climate Change Policies: Macroeconomic Effects, Structural Adjustments and Technological Change**

Rainer Walz, Joachim Schleich

New York:Springer, 2009. 170 pp. ISBN: 978-3-7908-2077-5, $119

**The Fluoride Wars: How a Modest Public Health Measure Became America’s Longest Running Political Melodrama**

R. Allan Freeze, Jay H. Lehr

Hoboken, NJ:John Wiley & Sons, 2009. 383 pp. ISBN: 978-0-470-44833-5, $39.95

**Twenty Years of Ozone Decline: Proceedings of the Symposium for the 20th Anniversary of the Montreal Protocol**

Christos Zerefos, Georgios Contopoulos, Gregory Skalkeas, eds.

New York:Springer, 2009. 470 pp. ISBN: 978-90-481-2468-8, $169

**Uncertainties in Environmental Modelling and Consequences for Policy Making**

Philippe Baveye, Jaroslav Mysiak, Magdeline Laba, eds.

New York:Springer, 2009. 401 pp. ISBN: 978-90-481-2635-4, $119

**Unquenchable: America’s Water Crisis and What to Do About It**

Robert Glennon

Washington, DC:Island Press, 2009. 416 pp. ISBN: 978-1-59726-436-5, $27.95

**Wetland Ecosystems**

William J. Mitsch, James G. Gosselink, Li Zhang, Christopher J. Anderson

Hoboken, NJ:John Wiley & Sons, 2009. 304 pp. ISBN: 978-0-470-28630-2, $75

